# Enhanced H3K4 Trimethylation in TNF-*α* Promoter Gene Locus with Cell Apoptosis in the Ventral-Medial Striatum following Opioid Withdrawal of Neonatal Rat Offspring from Morphine-Addicted Mothers

**DOI:** 10.1155/2021/9828995

**Published:** 2021-06-16

**Authors:** Pei-Ling Wu, Jau-Ling Suen, Chun-Hwa Yang, Kuang-Che Kuo, Yu-Chen S. H. Yang, San-Nan Yang

**Affiliations:** ^1^School of Medicine, College of Medicine, I-Shou University, Kaohsiung, Taiwan; ^2^Department of Pediatrics, E-Da Hospital, Kaohsiung, Taiwan; ^3^Graduate Institute of Medicine, College of Medicine, Kaohsiung Medical University, Kaohsiung, Taiwan; ^4^Department of Pediatrics, Kaohsiung Chang Gung Memorial Hospital and Chang Gung University College of Medicine, Kaohsiung, Taiwan; ^5^Joint Biobank, Office of Human Research, Taipei Medical University, Taipei, Taiwan

## Abstract

Prenatal opioid exposure might disturb epigenetic programming in the brain of neonatal offspring with various consequences for gene expressions and behaviors. This study determined whether altered trimethylation of histone 3 at lysine 4 (H3K4me3) in the promoter of the tumor necrosis factor-*α* (*tnf-α*) gene with neural cell apoptosis was involved in the ventral-medial striatum, an important brain region for withdrawal symptoms, of neonatal rat offspring from morphine-addicted mothers. Female adult rats were injected with morphine before gestation and until 14 days after giving birth. On postnatal day 14 (P14), rat offspring from morphine-addicted mothers were subjected to an opioid-withdrawal protocol and were analyzed 2 or 8 h after administration of that protocol. Expressions of the TNF-*α* protein, H3K4me3 in the *tnf-α* promoter gene, and neural cell apoptosis within the ventral-medial striatum of neonatal rat offspring were evaluated. In the absence of significant opioid withdrawal (2 h after initiation of the opioid-withdrawal protocol on P14), prenatal morphine exposure led to increased levels of H3K4me3 in the *tnf-α* promoter gene, of the TNF-*α* protein, and of neural cell apoptosis within the ventral-medial striatum of neonatal rat offspring. Following opioid withdrawal (8 h after initiation of the opioid-withdrawal protocol on P14), differential expression of H3K4me3 in the *tnf-α* promoter gene locus and upregulation of the level of TNF-*α* protein expression were further enhanced in these offspring. In addition, increased levels of caspase-3 and neural cell apoptosis were also observed. Taken together, this study revealed that prenatal opioid exposure can activate an epigenetic histone mechanism which regulates proinflammatory factor generation, which hence, led to cell apoptotic damage within the ventral-medial striatum of neonatal rat offspring from morphine-addicted mothers. More importantly, the opioid-withdrawal episode may provide augmented effects for the abovementioned alterations and could lead to deleterious effects in the neonatal brain of such offspring.

## 1. Introduction

Early life adversity is related to increased risks for developing many behavioral and psychiatric disorders [1]. A spectrum of prenatal, intrapartum, and postnatal episodes is likely to contribute to neurodevelopment via interactions with the individual's genotype to disrupt specific neural and psychophysiological system-related cognitive functions, thereby enhancing the risk for psychopathologies later in life [2, 3]. Indeed, children exposed to perinatal opioid drugs appear to have a higher mortality rate after birth and suffer from neurodevelopmental consequences and behavioral problems [4, 5].

During the fetal and neonatal periods, the central nervous system (CNS) displays highly significant plasticity and is vulnerable to changes by prenatal influences such as prenatal opioid exposure [6–8]. The mechanisms through which early life exposures play important roles for later-life psychopathologies remain undetermined, but an epigenetic mechanism represents a reasonable candidate [9, 10]. The ventral-medial striatum of the mammalian brain serves as an integration center to mediate goal-directed behaviors (i.e., rewards of drug craving and drug addiction) [11]. Previous studies demonstrated that morphine exposure can trigger the production of proinflammatory cytokines, including tumor necrosis factor- (TNF-) *α*, interleukin- (IL-) 1, and IL-6 [12–14]. Chronic opioid administration leads to increased messenger (m)RNA levels of IL-1 and IL-6 in the ventral-medial striatum of rats *in vivo* [15]. In addition, morphine withdrawal neuro-behaviors can induce TNF-*α* release from neural cells [13]. Recently, TNF-*α* expression levels were found to increase in the amygdala during morphine-withdrawal episodes [14]. Indeed, inflammation in the brain not merely occurs with direct opioid exposure and withdrawal but has transplacental effects on newborn offspring as well; for instance, offspring suffering from prenatal opioid exposure exhibited increased TNF-*α* production in the hippocampus [16]. Prenatal stresses, such as chronic illicit drug abuse, can dysregulate the maternal immune function and cause elevated proinflammatory cytokine levels in the brain of neonatal offspring [17]. Taken together, those studies revealed that prenatal opioid exposure can generate transplacental inflammation cascades during early life.

The epigenetic histone regulation in a target promoter gene locus might play a role in the molecular basis of mammalian drug-craving and addiction [18, 19]. Recently, trimethylation of histone H3 at lysine 4 (H3K4me3), one type of histone regulation, was suggested to act as a transcription booster for the *tnf-α* gene expression within the promoter region [18, 20]. In addition, early life stress or drug exposure can also induce histone modifications within the ventral tegmental area resulting in dysregulated signal conduction [21]. However, little is known about histone methylation as a result of perinatal opioid exposure with apoptosis in the offspring brain. Thus, the aim of this study was to examine the hypothesis that prenatal opioid exposure activates the trimethylation of H3K4me3 in the *tnf-α* promoter gene and is associated with neural cell apoptosis in the ventral-medial striatum of neonatal rat offspring (on postnatal day 14; P14).

## 2. Materials and Methods

### 2.1. Experimental Animal Protocol

Female adult Sprague-Dawley (SD) rats which underwent the opioid-withdrawal protocol were housed under a 12 h light/dark cycle with humidity maintained around 60% ~70% and were initially injected with morphine hydrochloride (2 mg/kg body weight (BW), subcutaneously twice daily with the dosage progressively increased at a rate of 1 mg/kg BW every 7 days with a maximum dose of 7 mg/kg BW) [6–8, 22]. These female rats were mated on about day 7 or 8. During pregnancy and after the rat offspring were born, morphine was still routinely administered as scheduled until the offspring were 14 days old. The last opioid injection was on postnatal day 13 (P13). Throughout the experimental course, there were no significant opioid withdrawal symptoms between the maternal opioid injections. The vehicle-control group consisted of maternal SD rats injected with NaCl at the same dose and interval as the experimental group.

We evaluated the growth of BW of rat offspring on postnatal day 7 (P7) and P14 (an age regarded as a neonate in the human lifespan) [23]. On P14, rat offspring from maternal rats were transferred to an observational chamber maintained at around 33°C with warm light and were observed for 2 h for cumulative episodes of and latency in exhibiting opioid-withdrawal behaviors, such as rearing, teeth chattering, backward locomotion, tremors, and wet-dog shaking. Rat offspring from morphine-addicted mothers were divided into two groups: an early group represented rat offspring observed for opioid withdrawal behaviors for 2 h immediately after transfer, after which they were sacrificed for biochemical experiments (the early group, *n* = 48 rats); and the late group represented rat offspring for observation of opioid-withdrawal behaviors for 2 h starting from the 6th hour after transfer. These rat offspring were then sacrificed for biochemical experiments (the late group, *n* = 48 rats). Rat offspring from the vehicle-control group were transferred to an observational chamber on P14 and were observed for opioid-withdrawal behaviors for 2 h starting from the 6th hour after transfer. These rat offspring were then sacrificed for biochemical experiments (the control group, *n* = 48 rats). There were no differences in sex among the three groups.

All experimental protocols were approved by the Animal Care and Use Committee at the National Defense Medical Center, Taiwan (NDMC-04-088 and IACUC-04-085) with all actions undertaken being designed to lessen suffering and decrease the number of animals used.

### 2.2. Preparation of Slices of the Ventral-Medial Striatum Brain Region

After the opioid-withdrawal protocol was completed on P14, the pups were sacrificed, and brain slices (400 *μ*m) containing the ventral-medial striatum brain region were immediately harvested with a vibro-slicer (Campden Instruments, Sileby, Loughboroug, UK). Brain slices were selectively transferred to an incubation chamber perfused with oxygenated artificial cerebrospinal fluid (95% O_2_/5% CO_2_ gas, at 30.0 ± 0.5°C) for 1 h. Artificial cerebrospinal fluid consisted of (in mM) NaCl (124), MgCl_2_ (1), CaCl_2_ (2), KCl (3.5), NaH_2_PO_4_ (1.25), NaHCO_3_ (26), and D-glucose (10) at pH 7.4 and with an osmolarity of 305 ± 5 mOsm.

### 2.3. Immunoblotting

Cells of the ventral-medial striatum were lysed in buffer (10 mM Tris at pH 7.4, 150 mM NaCl, 1 mM PMSF, 0.2% Triton X-100, 2 mM EDTA, and 1× protease inhibitor mixture). The protein concentration was clarified with a BCA assay (Thermo Scientific, Rockford, IL, USA). Cell lysates were sorted on sodium dodecylsulfate polyacrylamide gel electrophoresis (SDS-PAGE), later transferred to nitrocellulose membranes, and then probed with antibodies against TNF-*α* (SC-1351; Santa Cruz) and cleaved caspase-3 (no. 9662; Cell Signaling). Depending on the different types of primary antibody, either rabbit anti-mouse immunoglobulin G (IgG) or goat anti-rabbit IgG (1: 3000) was chosen as the secondary antibody. Immunoreactive proteins were detected using the BioSpectrum 810 Imaging System (UVP).

### 2.4. Real-Time Polymerase Chain Reaction (PCR)

Isolation of RNA, including synthesis of complementary (c)DNA, and DNase treatment was accomplished using the Transcriptor First Strand cDNA Synthesis Kit RT-PCR System™ (Roche, Indianapolis, IN, USA) following the manufacturer's instructions. The two-step real-time PCR with the LightCycler™ System (Roche) and Sybr Green I dye was used for monitoring the PCR. FastStart DNA Master SYBR Green I (Roche) was performed according to the producer's instructions and used for the real-time PCR. This study utilized the following set of primers: TNF-*α*: forward (5′-CCCTACGGGTCATTGAGAGA-3′) and reverse (5′-GGTTGTGGACTGCCTTTTGT-3′); and 18s ribosomal (r)RNA: forward (5′-CCAGTAAGTGCGGGTCATAA-3′) and reverse (5′-TAGTCAAGTTCGACCGTCTTC-3′). The thermal protocol of the PCR was set to 95°C (for 4 min), followed by 30 amplification cycles of 94°C (for 30 s), 62°C (for 1 min), and 72°C (for 1 min), with a final melting curve analysis. Amplicons were analyzed by the melting curve (Light Cycler software) and agarose gel electrophoresis; the LightCycler analytical system was applied to decide cycle threshold (CT) values, and the relative messenger (m)RNA level of each experiment was assessed by a real-time PCR followed by measurement by the relative CT method (*ΔΔ*CT) (the expression of the objective was normalized to an endogenous standard (18S rRNA) and compared to a calibrator (cDNA from a pooled sample)).

### 2.5. Chromatin Immunoprecipitation (ChIP) Assay

A ChIP assay was performed as previously described [24, 25]. Briefly, 5 × 10^5^ cells were treated with 1% formaldehyde at room temperature (for 10 min) accompanied by sonication of DNA and immunoprecipitation of chromatin overnight with antibodies of trimethylated H3K4 (2 *μ*g for 25 *μ*g of chromatin; antihistone H3K4me3 antibody, ChIP Grade; ab8580; Abcam) and later purification with a ChIP kit (no. 17-295; Upstate Biotechnology, Lake Placid, NY, USA). Probes and primers were planned by exploring the proximal promoter and intronic enhancer areas of the *tnf-α* gene [24, 25], including the following subregions as described below relative to the transcription start site: *tnf-α* 1 (-2686 to -2667); forward, 5′-CCCTAGTCCTCCTGGGATGT-3′ and reverse, 5′-GCCTGCTGCAACAGAGAGA-3′; *tnf-α* 2 (-2202 to -2183); forward, 5′-CGTCTCACTATGCCTGGGTCT-3′ and reverse, 5′-AAGCAAAGCACTTCTACCAAAT-3′; *tnf-α* 3 (-1672 to -1653); forward, 5′-AAACTCAGACCAGGCTGCAT-3′ and reverse, 5′-CAGGTCATCTCTTGACGTGGT-3′; *tnf-α* 4 (-502 to -480); forward, 5′-GAGTTCTGCATGTATTGGATAGG-3′ and reverse, 5′-TGCTACCAAGCCTAAAGACC-3′; and *tnf-α* 5 (-230 to -213); forward, 5′-GGTTCAGTTCCCAGCACCTA-3′ and reverse, 5′-ATGGGCATATCTGCACAGCA-3′. PCRs were conducted on the ABI Gene Amp ⓐ PCR System (Applied Biosystems). The quantity of immunoprecipitated DNA was calculated and compared to the quantity of total input DNA.

### 2.6. Apoptosis Evaluation

A double immunofluorescence investigation by laser-scanning confocal microscopy was applied to examine apoptosis in neurons of brain sections of the ventral-medial striatum, which were cut at a coronal plane and measured 30 *μ*m and then stained with an antineuronal nuclei (NeuN) antibody (clone A60, MAB377; Chemicon, Temecula, CA, USA) to identify neuronal cells in the presence or absence of terminal uridine nick-end labeling (TUNEL; 11684795910, Sigma-Aldrich, St. Louis, MO, USA), and immunostaining was used to detect DNA break-down. A Cy3-conjugated anti-mouse antibody for staining NeuN and a fluorescein-conjugated antibody for staining TUNEL were used for a secondary amplification that was visualized with a Zeiss LSM510 laser microscope (Thornwood, NY, USA). Quantitative analyses of the NeuN-sensitive cell density, TUNEL-sensitive cell density, and NeuN/TUNEL-sensitive cell density were performed in the ventral-medial striatum according to the brain atlas [26]. Average results for each group were taken from six slices per animal.

### 2.7. Statistical Analysis

Data are shown as mean ± standard error of the mean (SEM). Data were analyzed using SigmaPlot 10.0 and Sigmastat 3.5. A one-way analysis of variance (ANOVA) with Bonferroni's test for post hoc comparisons was applied with the level of significance set to *p* < 0.05.

## 3. Results

### 3.1. Opioid-Withdrawal Behaviors

**Figure 1:**
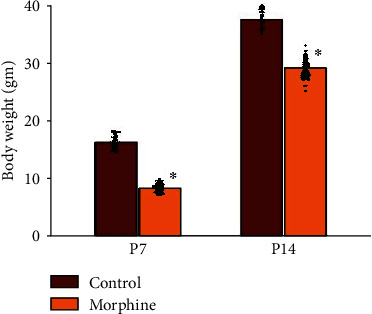
The BW of rat offspring of the prenatally opioid-exposure group was statistically significantly lower. The BW of rat offspring on P7 was as follows: the control group: 16.3 ± 1.3 g (*n* =48 rats) vs. the prenatally opioid-exposure group: 8.3 ± 0.7 g (*n* = 96 rats). And the BW of rat offspring on P14 was as follows: the control group: 37.6 ± 1.7 (*n* =48 rats) vs. the prenatally opioid-exposure group: 29.2 ± 1.6 g (*n* = 96 rats). Scatted dots represent the data points in each group. ^∗^*p* < 0.05, compared to the control group.

We observed whether prenatal opioid exposure disrupted the growth in BW after birth. In Figure 1, the BW of rat offspring of the prenatally opioid-exposure group was statistically significantly lower (P7: the prenatally opioid-exposure group: 8.3 ± 0.7 g, *p* < 0.05, *n* = 96 rats vs. the vehicle-control group: 16.3 ± 1.3 g, *n* = 48 rats; P14: the prenatally opioid-exposure group: 29.2 ± 1.6 g, *p* < 0.05, *n* = 96 rats vs. the vehicle-control group: 37.6 ± 1.7, *n* = 48 rats).

**Figure 2:**
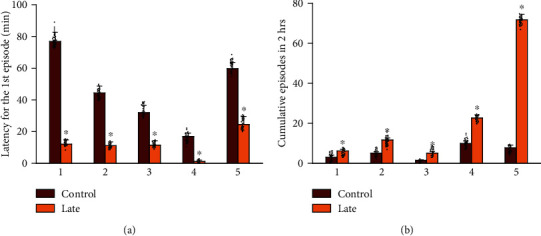
Observations of opioid-withdrawal behaviors in rat offspring on P14. (a) The rat offspring which were observed for 2 h starting from the 6th hour after transfer (the late group) exhibited shorter latency in exhibiting opioid-withdrawal behaviors. (b) There were more opioid-withdrawal behaviors in a 2 h period in the late group. 1: rearing; 2: teeth chattering; 3: backward locomotion; 4: tremor; 5: wet-dog shaking. Each group was derived from independent 48 rats, respectively. ^∗^*p* < 0.05, compared to the control group.

Rat offspring in the early group did not express significant opioid-withdrawal behaviors, compared to the control group (*p* > 0.05, *n* = 48 rats in each group, data not shown). In contrast, as shown in Figure 2, the late group exhibited shorter latency in exhibiting opioid-withdrawal behaviors (e.g., rearing, teeth chattering, backward locomotion, tremors, or wet-dog shaking) and had more opioid-withdrawal behaviors in a 2 h period, compared to rat offspring born from the control group at the 6th hour after transfer (*p* < 0.05, *n* = 48 rats in each group).

### 3.2. Prenatal Opioid Exposure and TNF-*α* Expression

**Figure 3:**
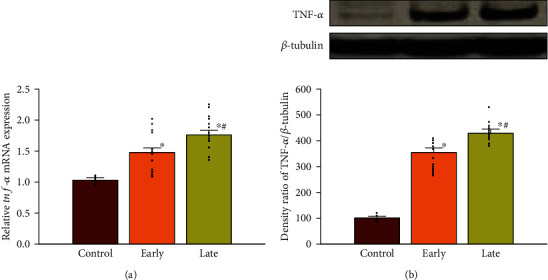
Prenatal opioid exposure enhanced the TNF-*α* expression in the ventral-medial striatum of neonatal rat offspring on P14. (a) The expression of TNF-*α* at mRNA level in the ventral-medial striatum of neonatal rat offspring from morphine-addicted mothers. (b) The expression of TNF-*α* at protein level in the ventral-medial striatum of neonatal rat offspring from morphine-addicted mothers. The groups were as follows: the control group (*n* = 12 rats), the early group (*n* = 12 rats), and the late group (*n* = 12 rats). The upper panel indicates representative immunoblots. *β*-Tubulin served as an internal standard control and was not significantly changed across lanes. Scatted dots represent the data points in each group. ^∗^*p* < 0.05, compared to the control group. #*p* <0.05, compared to the early group.

Figure 3 shows levels of TNF-*α* mRNA and protein in the ventral-medial striatum of neonatal rat offspring (P14) from morphine-addicted mothers. There were significant increases at the mRNA and protein levels of the TNF-*α* expression in the early group compared to the control group (*p* < 0.05, *n* = 12 rats in each group). In addition, mRNA and protein levels of the TNF-*α* expression were further enhanced in the late group compared to the early group (*p* < 0.05, *n* = 12 rats in each group).

### 3.3. Prenatal Opioid Exposure and H3K4me3 Modifications in the *tnf*-*α* Promoter

**Figure 4:**
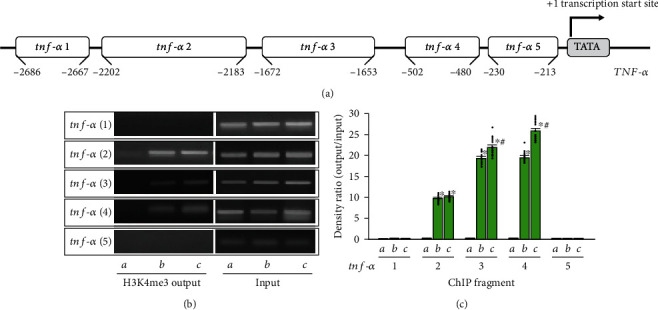
Prenatal opioid exposure activated differential enhancement of H3K4me3 in the *tnf*-*α* promoter gene locus in the ventral-medial striatum. (a) The figure demonstrated the subregions relative to the transcription start site of *tnf-α* promoter gene locus as follows: *tnf-α* 1 (−2667 to −2686), *tnf-α* 2 (−2183 to −2202), *tnf-α* 3 (−1653 to −1672), *tnf-α* 4 (−174 to −496), and *tnf-α* 5 (−205 to −224). (b) The panel illustrations indicate representative ChIP assay of the levels of trimethylated H3K4 in the *tnf-α* promoter gene locus at the subregions. The insets *a*, *b*, and *c* are as follows: the control group (*n* = 12 rats), the early group (*n* = 12 rats), and the late group (*n* = 12 rats). (c) The effects of prenatal opioid exposure in trimethylated H3K4 levels in the *tnf-α* promoter gene is summarized in three groups as described above. The DNA input signal served as the internal loading control and was not significantly different across lanes. Scatted dots represent the data points in each group. ^∗^*p* < 0.05, compared to the control group. #*p* < 0.05, compared to the early group.

To determine whether activation of H3K4me3 in the *tnf*-*α* promoter gene was associated with increased levels of TNF-*α* as seen in Figure 3, ChIP analyses using PCR primers matching five subregions of the *tnf-α* promoter gene were performed in the ventral-medial striatum of neonatal rat offspring. As shown in Figure 4, prenatal opioid exposure increased levels of H3K4me3 in subregions 2, 3, and 4 of the *tnf-α* promoter gene (*p* < 0.05, *n* = 12 rats in each group). Furthermore, in the late group, there were further increased levels of H3K4me3 activity in subregions 3 and 4 of the *tnf-α* promoter gene locus compared to the early group (*p* < 0.05, *n* = 12 rats in each group).

### 3.4. Prenatal Opioid Exposure and Apoptotic Caspase-3 Activity

**Figure 5:**
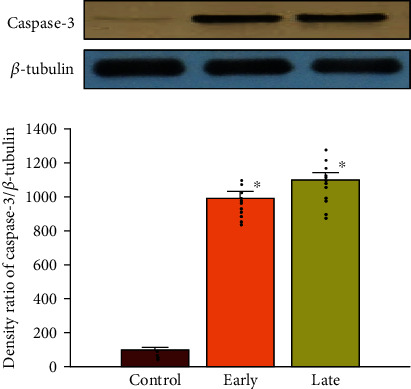
Prenatal opioid exposure enhanced the level of the caspase-3 expression in the ventral-medial striatum of neonatal rat offspring on P14. The level of cleaved caspase-3 in the ventral-medial striatum of neonatal rat offspring from morphine-addicted mothers is summarized in three groups as follows: the control group (*n* = 12 rats), the early group (*n* = 12 rats), and the late group (*n* = 12 rats), respectively. The upper panel reveals illustrative immunoblots. *β*-Tubulin was used as an internal standard control and was not significantly different across lanes. Scatted dots represent the data points in each group. ^∗^*p* < 0.05, compared to the control group. #*p* < 0.05, compared to the early group.

To observe whether prenatal opioid exposure caused the abovementioned TNF-*α* enhancement combined with cell apoptosis, cleaved caspase-3 activity was evaluated in the ventral-medial striatum of neonatal rat offspring (P14) from morphine-addicted mothers. As shown in Figure 5, there was an increased level of the cleaved caspase-3 expression in the early group compared to the control group (*p* < 0.05). In addition, the level of cleaved caspase-3 expression in the late group did not statistically differ compared to the early group (*p* > 0.05, *n* = 12 rats in each group).

### 3.5. Prenatal Opioid Exposure and Neural Cell Apoptosis

**Figure 6:**
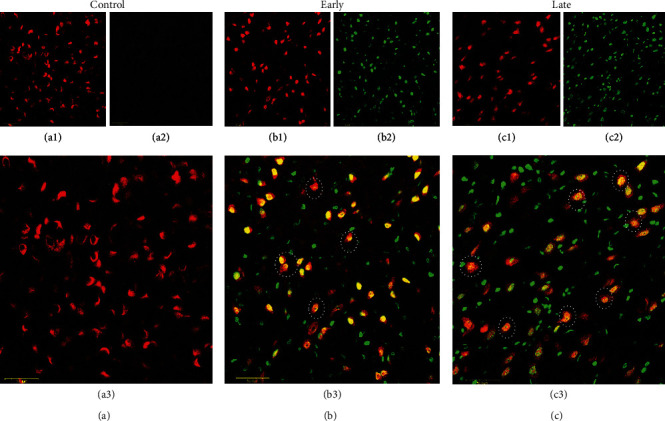
Prenatal opioid exposure induced cell apoptosis in the ventral-medial striatum of neonatal rat offspring (P14). Colocalization of NeuN- (red for neuron identification) and TUNEL- (green for apoptotic cells) sensitive cells was recognized by double immunofluorescence staining with laser-scanning confocal microscopy. The NeuN-sensitive cells (panel 1), TUNEL-sensitive cells (panel 2), and the combined pictures of the NeuN- and TUNEL-sensitive cells (panel 3) are as follows: the control (a1–a3), the early group (b1–b3), and the late group (c1–c3), respectively. The dotted circles reveal representative apoptotic neurons. Bar = 50 *μ*m.

**Figure 7:**
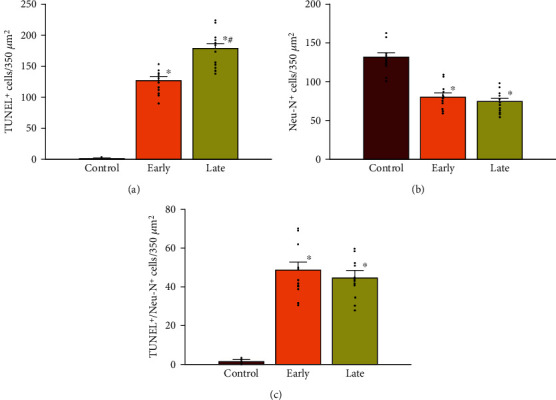
Prenatal opioid exposure induced cell apoptosis in the ventral-medial striatum of neonatal rat offspring (P14). The summarized data of TUNEL-sensitive cells (a), NeuN-sensitive cells (b), and colocalizations of TUNEL/NeuN-sensitive cells (c), for counted ventral-medial striatum are as follows: the control group, the early group, and the late group, respectively. Average results for each group were taken from six slices per animal (*n* = 12 rats in each group). Scatted dots represent the data points in each group. ^∗^*p* < 0.05, compared to the control group. #*p* < 0.05, compared to the early group.

To confirm the increased levels of the above-described caspase-3 activity, confocal laser-scanning microscopy was applied to study neonatal rat offspring (P14) from morphine-addicted mothers. Figures 6(a)–6(c) are representative morphological illustrations, and Figures 7(a)–7(c) demonstrate summarized data. As indicated in Figure 7(a), there was an increased number of TUNEL-sensitive cells within the ventral-medial striatum in the early group compared to the control group (*p* < 0.05, *n* = 12 rats in each group). In addition, a further increase in TUNEL-sensitive cells was observed in the late group compared to the early group (*p* < 0.05, *n* = 12 rats in each group) (Figure 7(a)).  Figure 7(b) shows that prenatal opioid exposure led to fewer NeuN-sensitive cells in the early group compared to the control group (*p* < 0.05). The number of NeuN-sensitive cells in the late group did not statistically differ compared to the early group (*p* > 0.05, *n* = 12 rats in each group) (Figure 7(b)). In Figure 7(c), the number of cells displaying colocalization of NeuN and TUNEL responses within the ventral-medial striatum was significantly higher in neonatal rat offspring (P14) from morphine-addicted mothers compared to the control group (*p* > 0.05, *n* = 12 rats in each group). These results reveal that prenatal opioid exposure can induce neural cell apoptosis in the ventral-medial striatum of neonatal rat offspring from morphine-addicted mothers.

## 4. Discussion

The major findings of this study revealed that prenatal opioid exposure activated differential trimethylation of H3K4 modifications in the promoter locus of the *tnf*-*α* gene and was associated with cell apoptosis in the ventral-medial striatum of neonatal rat offspring from morphine-addicted mothers, suggesting possible early life adversity within the ventral-medial striatum of the mammalian brain.

Previous studies suggested that epigenetic histone modifications regulate gene expressions through a specific site over a promoter in response to various stimuli [27]. However, most studies evaluated global changes of epigenetic modifications rather than adopting an approach to investigate a specific gene. Previous studies indicated a higher possibility of DNA methylation located in specific loci of the *tnf-α* promoter gene as detected from human gingiva with periodontitis [28, 29]; additionally, exposure to lipopolysaccharide induced different but focused regions of histone modifications including methylation in the promoter region of the *tnf-α* gene [27]. This study further disclosed the differential enhancement of H3K4me3 modifications in the *tnf-α* promoter gene locus of the ventral-medial striatum of neonatal rat offspring from morphine-addicted mothers, and this enhancement over a different promoter locus of the *tnf*-*α* gene implies that respective transcriptional factors can be recruited into transcription and become affected by prenatal opioid exposure. For example, signal transducer and activator of transcription 6 (STAT6) (located in the *tnf-α* 2 subregion) were reported to mediate neuroinflammation caused by ethanol in mice with a traumatic brain injury [30]. Engrailed homeobox 1 (En1) (located in the *tnf*-*α* 3 subregion) and pituitary homeobox 3 (Pitx3) (located in the *tnf*-*α* 4 subregion) are involved in dopaminergic neuronal development, which is related to drug addiction and reward behaviors [31]. In this study, we demonstrated increased levels of H3K4me3 in subregions 2, 3, and 4 of the *tnf*-*α* promoter gene in the ventral-medial striatum of neonatal rat offspring from morphine-addicted mothers (Figure 4), accompanied by increased levels of TNF-*α* mRNA and protein (Figure 3). These results hint at possible alterations in activation of transcription factors responsible for proinflammatory factor generation (e.g., TNF-*α*). However, which transcription factors are involved and are affected by epigenetic histone modifications in offspring due to prenatal opioid exposure are still unclear.

Epigenetic histone modifications persistently appear not only during acute or chronic exposure but are long-lasting as well, even after withdrawal episodes [32, 33]. Methamphetamine increased the expression of H3K4me3 during addiction in the dorsal striatum of animals, and it was globally expressed even after termination of methamphetamine exposure [34]. Similar, prenatal opioid exposure increased levels of H3K4me3 in the *tnf*-*α* promoter gene in this study. And as the late group presented opioid-withdrawal behaviors, there was a further enhancement of H3K4me3 activity in specific subregions. Taken together, epigenetic histone modifications seem to be related to mediation of priming or desensitization of specific genes in illicit drug exposure, and such epigenetic histone modifications can be latent, while persistently altering the chromatin structure after withdrawal episodes [35]. However, we mainly evaluated the H3K4me3 expression in the ventral-medial striatum from neonatal rat offspring in the acute stage of prenatal morphine exposure and/or withdrawal in this study, which might mimic the clinical state of neonatal abstinence syndrome. Future studies should also investigate histone modifications at additional time points in order to evaluate the long-term effects of prenatal opioid exposure.

Enhancement of TNF-*α* production was associated with cell apoptosis within the ventral-medial striatum by prenatal opioid exposure which increases the neurobiological basis of risks for neuropsychiatric disorders in later life. Such enhanced TNF-*α* release indicates an early proinflammatory response leading to activation of apoptotic pathways such as caspase-3 signaling [36–38]. However, in this study, there was no significant decrease in apoptotic neurons in the late group (Figure 7). Indeed, morphine can directly induce apoptosis of microglia and neurons *in vitro*, but it seemed that chronic morphine exposure might not affect glial cells at an early age *in vivo* from animal models [36, 37]. Therefore, apoptosis did play a role in prenatal opioid exposure, but the detailed involvement of specific cell types remains to be investigated. In this study, prenatal opioid exposure and withdrawal induced TNF-*α* production might lead to neural cell apoptosis in the ventral-medial striatum. In addition, opioid exposure might also enhance apoptosis in selected, specific brain areas, such as the amygdala, ventral-medial striatum, or prefrontal cortex *in vivo* [36, 38]. Prenatal heroin exposure enhances apoptosis in the hippocampus and results in impairments to learning and memory [39]. Those results indicate that prenatal opioid exposure and withdrawal might induce apoptosis in other specific brain areas and result in neurological impairments.

Recently, a closely interactive and related relationship between inflammation and neuropsychiatric disorders was proposed in the mammalian brain [40]. For instance, proinflammatory cytokines can affect cognition and mood behaviors by promoting neuroexcitatory reactions and impairing neuron plasticity [41]. Furthermore, as shown in this study, the epigenetic H3K4me3 mechanism for the upregulation of the TNF-*α* expression was associated with increased neural cell apoptosis in the ventral-medial striatum, which might contribute to neurological impairments. Such neural cell apoptotic damage within the ventral-medial striatum during early life might lead to increased dose requirements for opioid drugs to satisfy the need to increase the dosage and to fulfill drug-craving and addictive behaviors later in life. In addition, prenatal opioid exposure may initiate long-lasting alterations in the gene expressions of the synapse structure in the ventral-medial striatum in rat offspring [8, 22]. Such effects of prenatal opioid exposure seem to exist longer than expected. Our current results showed that prenatal opioid exposure induced neural cell apoptosis within the ventral-medial striatum in early life. However, whether neural cell apoptosis persistently appears in later life or the next generation is still unknown, and this has motivated us to conduct further experiments on the long-term effect of prenatal opioid exposure and withdrawal.

## 5. Conclusions

In summary, this study revealed that prenatal opioid exposure can activate an epigenetic histone mechanism for regulating proinflammatory factor generation and hence lead to cell apoptotic damage within the ventral-medial striatum of neonatal rat offspring from morphine-addicted mothers. More importantly, the opioid-withdrawal episode may provide augmented effects for such abovementioned alterations and may lead to adverse effects in the neonatal brain of these offspring.

## Data Availability

The data used to support the findings of this study are available from the corresponding author upon request.
